# Magnitude of the Benefit of Progression-Free Survival as a Potential Surrogate Marker in Phase 3 Trials Assessing Targeted Agents in Molecularly Selected Patients with Advanced Non-Small Cell Lung Cancer: Systematic Review

**DOI:** 10.1371/journal.pone.0121211

**Published:** 2015-03-16

**Authors:** Katsuyuki Hotta, Yuka Kato, Natasha Leighl, Nagio Takigawa, Rabab Mohamed Gaafar, Hiroe Kayatani, Taizo Hirata, Kadoaki Ohashi, Toshio Kubo, Masahiro Tabata, Mitsune Tanimoto, Katsuyuki Kiura

**Affiliations:** 1 Department of Hematology and Oncology, Okayama University Hospital, Okayama, Japan; 2 Department of Respiratory Medicine, Okayama University Hospital, Okayama, Japan; 3 Department of Medical Oncology and Hematology, University Health Network, Princess Margaret Hospital Division and the University of Toronto, Toronto, Ontario, Canada; 4 Department of General Internal Medicine 4, Kawasaki Medical School, Okayama, Japan; 5 National Cancer Institute, Cairo University, Cairo, Egypt; Kyushu University Faculty of Medical Science, JAPAN

## Abstract

**Background:**

In evaluation of the clinical benefit of a new targeted agent in a phase 3 trial enrolling molecularly selected patients with advanced non-small cell lung cancer (NSCLC), overall survival (OS) as an endpoint seems to be of limited use because of a high level of treatment crossover for ethical reasons. A more efficient and useful indicator for assessing efficacy is needed.

**Methods and Findings:**

We identified 18 phase 3 trials in the literature investigating EGFR-tyrosine kinase inhibitor (TKIs) or ALK-TKIs, now approved for use to treat NSCLC, compared with standard cytotoxic chemotherapy (eight trials were performed in molecularly selected patients and ten using an “all-comer” design). Receiver operating characteristic analysis was used to identify the best threshold by which to divide the groups. Although trials enrolling molecularly selected patients and all-comer trials had similar OS-hazard ratios (OS-HRs) (0.99 vs. 1.04), the former exhibited greater progression-free survival-hazard ratios (PFS-HR) (mean, 0.40 vs. 1.01; *P*<0.01). A PFS-HR of 0.60 successfully distinguished between the two types of trials (sensitivity 100%, specificity 100%). The odds ratio for overall response was higher in trials with molecularly selected patients than in all-comer trials (mean: 6.10 vs. 1.64; *P*<0.01). An odds ratio of 3.40 for response afforded a sensitivity of 88% and a specificity of 90%.

**Conclusion:**

The notably enhanced PFS benefit was quite specific to trials with molecularly selected patients. A PFS-HR cutoff of ∼0.6 may help detect clinical benefit of molecular targeted agents in which OS is of limited use, although desired threshold might differ in an individual trial.

## Introduction

Platinum-based and single-agent cytotoxic chemotherapies have been the standard treatments for advanced non-small cell lung cancer (NSCLC) patients in first-line and salvage settings, respectively [1–5]. Unfortunately, even upon application of such standard therapies, nearly all patients with advanced NSCLC experience disease progression, and thus, the ultimate goals of palliative chemotherapy are to prolong overall survival (OS) and to improve symptoms and the quality of life, rather than to cure. To date, evaluation of the efficacies of treatment strategies and the approval of most new agents used to treat advanced NSCLC have been based principally on OS prolongation in randomized clinical trials [6]. OS, the time from randomization to death from any cause, represents a direct measure of clinical benefit to the patient, and to date, no other endpoint has been shown statistically to serve as a suitable surrogate for OS in advanced NSCLC [7–12].

Many molecular targeted agents, including epidermal growth factor receptor (EGFR)- and anaplastic lymphoma kinase (ALK)-tyrosine kinase inhibitors (TKIs), have been assessed in phase 3 trials, compared with standard cytotoxic chemotherapy. Several trials have failed to demonstrate significant improvement in OS and/or progression-free survival (PFS), mainly because appropriate patient selection was not applied, i.e. the trials used molecularly unselected or all-comer designs. However, even in trials using only patients selected after evaluation of EGFR-mutant or ALK-fusion status (thus, molecularly selected patients) [13–21], EGFR- and ALK-TKIs failed to demonstrate any significant advantage in terms of OS, although these drugs are now widely approved in the U.S., E.U., and Japan. This would be explained by the inevitable high levels of crossover, essential from an ethical viewpoint, that allow control-arm patients to access these highly active investigational agents [13,18]. Thus, the data do not reflect inappropriate patient numbers or inadequate efficacy of the tested agents.

Currently, medical oncologists have strong views that in trials with crossover designs in molecularly selected patients, 1) the lack of any observed effect on OS does not necessarily mean that the agent is not efficacious; 2) the use of OS as the primary endpoint is limited; and 3) other endpoints are now urgently required to evaluate the efficacy of molecular targeted agents. If a significantly impressive benefit in PFS or overall response is evident specifically in molecularly-selected but not all-comer trials, we hypothesized that the PFS-hazard ratio (HR) or the odds ratio for the overall response would serve as a useful novel indicator in the former trial setting [10]. In order to propose novel efficient and useful markers of efficacy in this setting, we reviewed published phase 3 trials that compared EGFR- or ALK-TKIs (gefitinib, erlotinib, afatinib, or crizotinib) with traditional cytotoxic chemotherapy. We next identified differences in the magnitudes of PFS-HRs or odds ratios for the overall response in the two types of trials, those conducted in molecularly selected populations versus those conducted in all-comers.

## Methods

### Literature search

We performed a literature search of trials published between January 2003 and June 2014. To avoid publication bias, both published and unpublished trials were identified using a computer-based search of the PubMed database and of abstracts from conferences of the American Society of Clinical Oncology (ASCO), the European Society for Medical Oncology (ESMO), and the International Association for the Study of Lung Cancer (WCLC). The following search terms were used: “lung cancer AND advanced AND phase III study OR phase 3 study OR phase 3 trial OR phase III trial OR randomized controlled trial OR clinical trial OR controlled clinical trial”. Our search was also guided by a thorough examination of the reference lists of original and review articles, books, and meeting abstracts (ASCO, ESMO, and WCLC), and of the Physician Data Query registry of clinical trials.

### Trial selection

**Fig 1:**
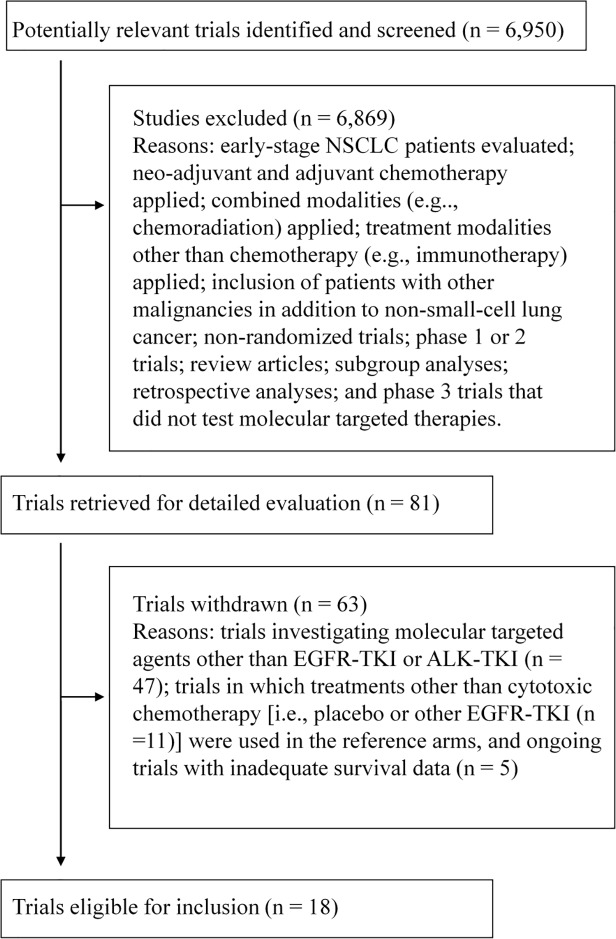
A flow chart demonstrating the selection process of the trials analyzed. NSCLC = non-small cell lung cancer, EGFR-TKI = epidermal growth factor receptor-tyrosine kinase inhibitor, ALK-TKI = anaplastic lymphoma kinase-tyrosine kinase inhibitor.

Eligible phase 3 trials were those that evaluated EGFR-TKIs or ALK-TKIs in the treatment of advanced NSCLC (Fig. 1), provided data on PFS, the overall response rate, and OS. Drugs acting on known specific molecular targets were defined as molecular targeted agents [22,23]. Trials designed to assess combined modality treatment, including radiotherapy and/or surgery, were excluded. We selected phase 3 trials that compared EGFR- or ALK-TKIs with existing cytotoxic chemotherapy.

### Data extraction

To avoid bias, two certified medical oncologists (K.H. and Y.K.) independently abstracted the trial data and compared their results, as described previously [5,24]. The following information was obtained from each report: year of trial initiation, number of patients randomized, treatment regimens, line of treatment, publication type, primary endpoint, PFS- and OS-HRs, and the number of responders. All data were verified for internal consistency, and disagreements were resolved by discussion between the investigators. The principal investigators of the trials were contacted and invited to confirm or update published data.

### Statistical analysis

We performed linear regression analysis to investigate associations after assigning weights determined by sample size to each trial. The strength of each association was defined *a priori* using the commonly accepted criterion of the coefficient of determination (the R-square value; r^2^) [25], which ranges from 0 < r^2^ < 1, with a higher score indicating a stronger association [24,25].

Any influence of trial design (molecularly selected patients vs. all-comers) on the PFS-HR or the odds ratio of the overall response was evaluated by multiple stepwise regression analysis using the following stepping criteria: *P*-value allowing model entry, ≤ 0.05; *P*-value compelling removal from the model, ≥ 0.20, with adjustment for several confounders including the year of trial initiation, line of treatment, primary endpoint, number of randomized patients, and type of reporting.

The significance of differences between groups was assessed using *t*-tests. Receiver operating characteristic (ROC) analysis was used to identify the most accurate discrimination thresholds dividing the groups. The most suitable cutoff level was defined as that closest to the top-left corner. The odds ratio for the overall response was calculated as follows: ([number of patients in the investigational arm who achieved a complete or partial response: A]/[number of randomized patients allocated to the investigational arm—A])/([number of patients in the control arm who achieved a complete or partial response: B]/[number of randomized patients allocated to the control arm—B]).

All *P* values were calculated using two-sided tests, and the level of significance was set at *P* < 0.05. Statistical analyses were performed using STATA (Version 11; StataCorp, Dallas, TX, USA).

## Results

### Trial demographics

Of the 6,950 trials screened, 18 phase 3 trials that investigated four molecular targeted agents (gefitinib, erlotinib, afatinib, and crizotinib) in patients with advanced NSCLC were identified (S1 Table). The trials included a total of 7,633 randomized patients (Fig. 1). The trial characteristics are listed in Table 1. We found eight trials enrolling molecularly selected patients and 10 all-comer trials. Sixteen trials evaluated EGFR-tyrosine kinase inhibitors (TKIs) in patients with EGFR-mutant NSCLC, and the remaining two trials assessed the use of crizotinib, an ALK-TKI, to treat ALK-rearranged NSCLC.

**Table 1:** Trial demographics (n = 18).

*Trial characteristics*			
	No. of randomly assigned patients per trial [(median (range)]		328 (161–1,466)
	Year of trial initiation [(median (range)]		2007 (2003–2011)
	Publication type (full text/abstract only)		16/2
	Primary endpoint (OS/PFS/TTP)		5/12/1
	First-line setting (y/n)		10/8
*Type of molecular targeted agent investigated*			
	EGFR-tyrosine kinase inhibitor		
		gefitinib	7
		erlotinib	6
		afatinib	3
	ALK-tyrosine kinase inhibitor		
		crizotinib	2

OS, overall survival; PFS, progression-free survival; TTP, time to progression; EGFR, epidermal growth factor receptor; ALK, anaplastic lymphoma kinase

### Correlation between the OS-HR and PFS-HR and between the OS-HR and the odds ratio of the overall response

**Fig 2:**
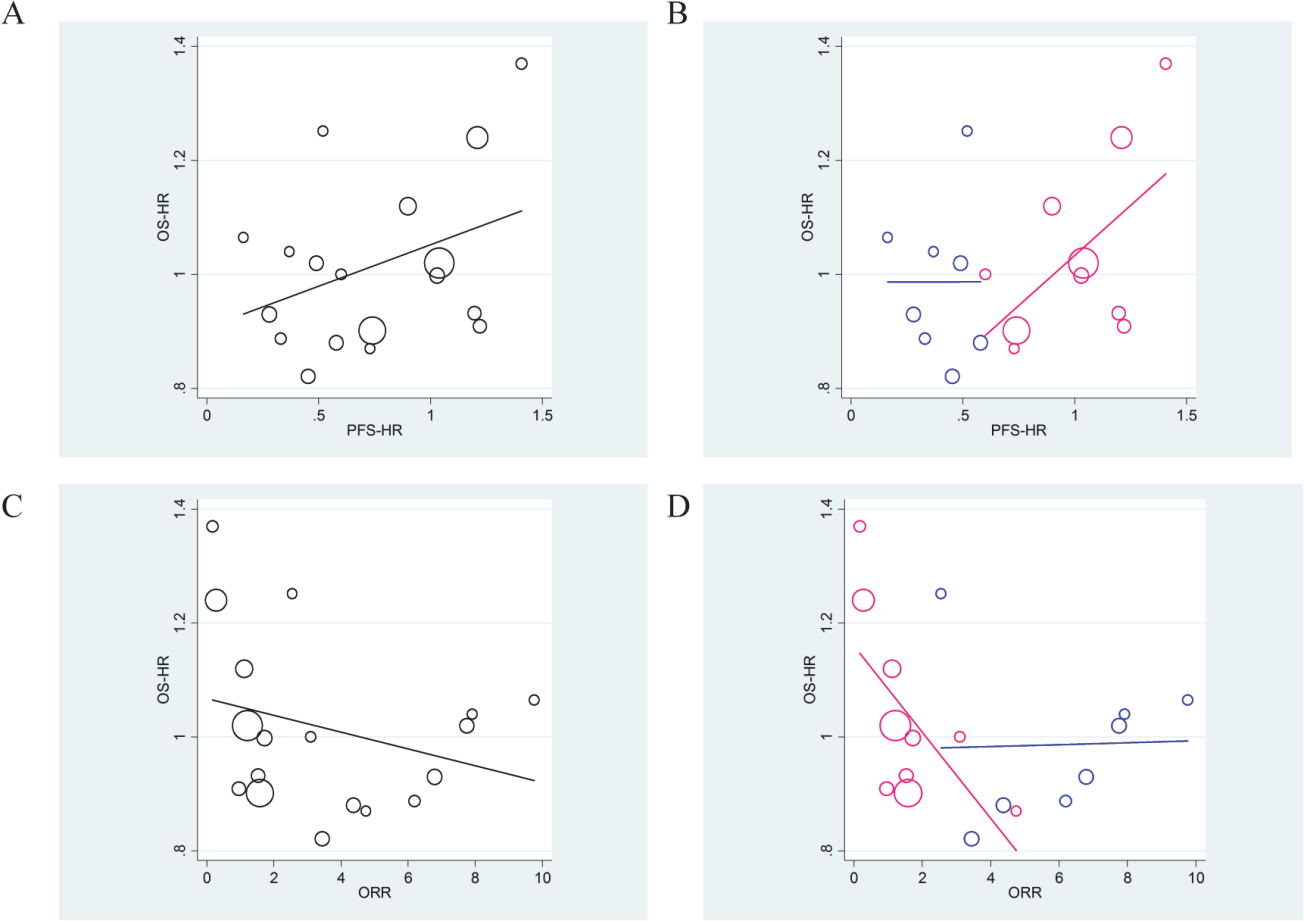
Associations between the progression-free survival-hazard ratio (PFS-HR) and overall survival (OS)-HR (R-squared = 0.233) (A), and that after stratification by trial design (B) (the molecularly selected patient design [blue], R-squared = 0.002, vs. the all-comer design [pink], R-squared = 0.409; *P*-value for interaction = 0.34). Associations between the odds ratio of the overall response and OS-HR (R-squared = 0.101) (C), and that after stratification by trial design (D) (the molecularly selected patient design [blue], R-squared = 0.039, vs. the all-comer design [pink], R-squared = 0.429; *P*-value for interaction = 0.03). All analyses were weighted by trial size.

First, we examined the strength of the correlation between the PFS-HR and the OS-HR. As shown in Fig. 2A, the PFS-HR had no meaningful association with OS-HR (overall R-squared value = 0.233), suggesting that the PFS-HR explained only 23.3% of the overall variability in OS-HR (Fig. 2A). This weak association was especially apparent in molecularly selected patient trials compared with all-comer design trials (R-squared values = 0.002 vs. 0.409, respectively; Fig. 2B). Similar observations were made when the association between the odds ratio of the overall response and the OS-HR were compared (overall R-squared value = 0.101, Fig. 2C). The association was more marked in trials with molecularly selected patients (R-squared values = 0.039 vs. 0.429, respectively; Fig. 2D).

Neither the PFS-HR nor the odds ratio of the overall response accurately predicted OS when a linear regression model was used to analyze data from molecularly selected patient trials.

### OS-HRs in trials with molecularly selected patients and all-comers designs

**Fig 3:**
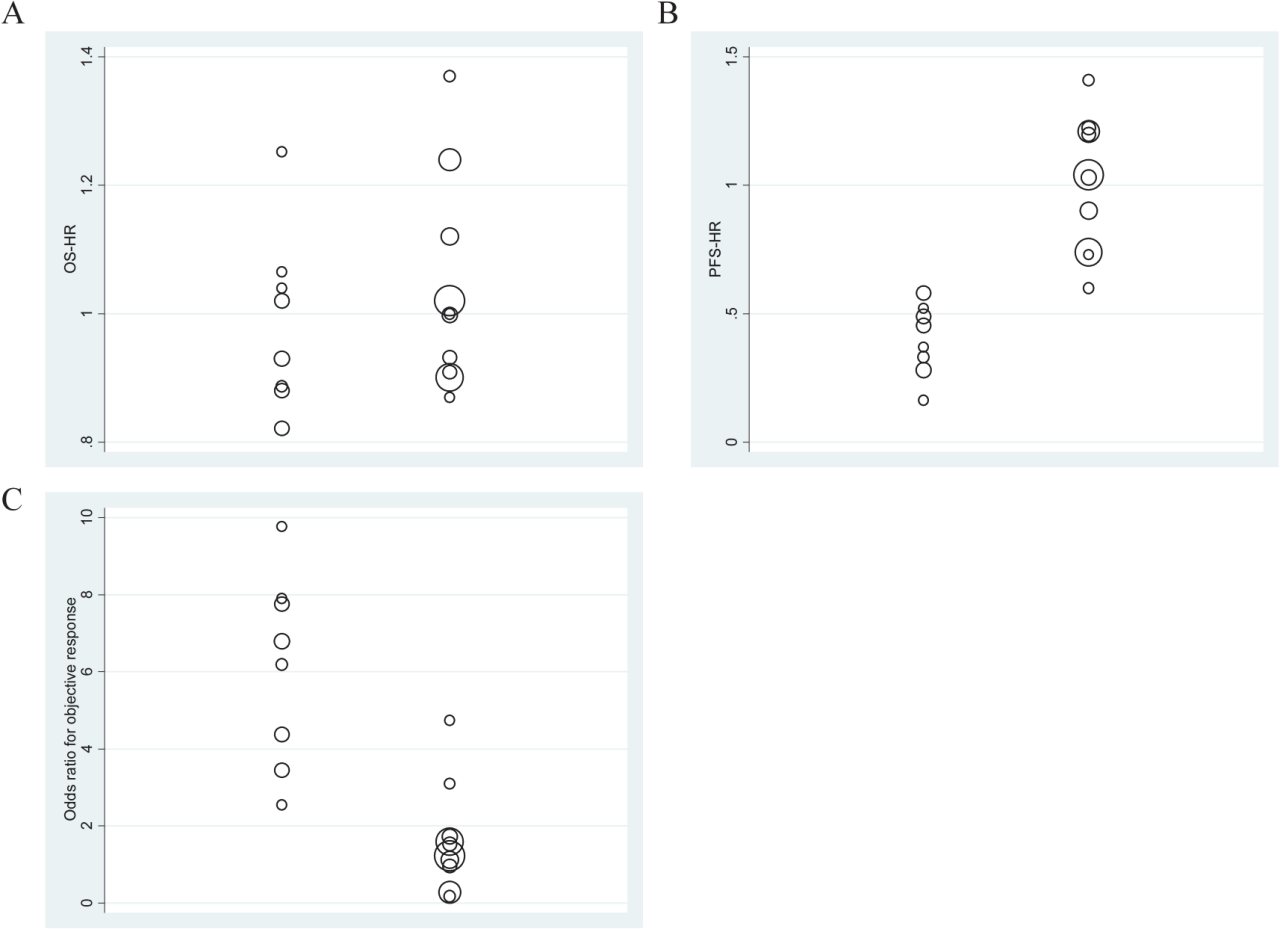
Distributions of hazard ratios (HRs) for overall survival (OS) (A) and progression-free survival (PFS) (B) and odds ratios for the overall response (C), stratified according to the two types of trials.

We found no significant difference in the OS-HRs between the two trial types (mean, 0.99 vs. 1.04 in molecularly selected patient trials vs. all-comer trials, respectively; *P* = 0.50) (Fig. 3A). In contrast, median survival time in molecularly selected patient trials was approximately double that in all-comer trials (median 23.1 and 26.6 months in the investigational and control arms of molecularly selected trials, respectively, compared with 11.9 and 12.2 months, respectively, in all-comer trials).

The left and right columns in each panel represent data from molecularly selected patient trials and all-comer trials, respectively. The diameter of each circle is representative of the size of the trial.

A, Trials with molecularly selected patients had almost identical OS-HRs, compared with those of all-comer trials (mean, 0.99 vs. 1.04, *P* = 0.50). B, The PFS-HRs were 0.40 vs. 1.01 in the two trial types (*P* < 0.01). C, Trials with molecularly selected patients had significantly greater odds ratios in terms of the overall response (mean; 6.10 vs. 1.64, *P* < 0.01).

### PFS-HRs in both molecularly selected patient and all-comer trials

We next investigated differences in the PFR-HRs between the two trial groups. Molecularly selected patient trials had a greater PFS-HR than did all-comer trials (mean, 0.40 vs. 1.01; *P* < 0.01; Fig. 3A). This significant influence of trial design on PFS-HR was observed even when several potential confounders were adjusted upon multivariate analysis; trials using molecular selection had a PFS-HR score 0.42 points lower than that of the all-comer trials; *P* < 0.01; Table 2).

**Table 2:** Multiple stepwise linear regression analysis of PFS-HR and the odds ratio for overall response.

		PFS-HR		Odds ratio for overall response	
**Factor**		Regression coefficient (95% CI)	*P*	Regression coefficient (95% CI)	*P*
	Trial design (molecularly-selected vs. all-comer)	−0.42 (−0.67 to −0.18)	< 0.01	4.46 (2.52–6.39)	< 0.01
	Primary endpoint (PFS vs. other)	−0.31 (−0.56 to −0.53)	0.02	excluded	
	Line of treatment (1^st^ vs. other)	Excluded		excluded	
	Year of trial initiation (before or in 2006 vs. 2007 or later)†	Excluded		excluded	
	No. of randomized patients	Excluded		excluded	
	Type of reporting (full text vs. abstract only)	Excluded		excluded	

The threshold p values for entry into and removal from the model were 0.05 and 0.20, respectively.

†The cutoff level was set as the median year of trial initiation. PFS-HR, progression-free survival-hazard ratio; CI, confidence interval.

**Fig 4:**
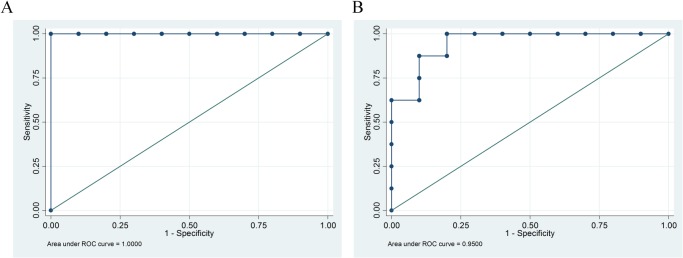
Receiver operating characteristic (ROC) curve defining cutoff levels predicting outcomes in eligible trials with molecularly selected patients in terms of the progression-free survival-hazard ratio (PFS-HR) (A) and the odds ratio for the overall response (B). The most suitable cutoff was defined as that closest to the upper left corner. A, A PFS-HR of 0.60 was the optimal cutoff for distinguishing molecularly selected patient trials from all-comer trials (sensitivity 100%, specificity 100%, and AUC [area under the receiver operating characteristic curve], 1.00). B, The odds ratio for an overall response of 3.40 was a potentially useful cutoff to distinguish trials with molecularly selected patients from all-comer trials (sensitivity 88%, specificity 90%, and AUC = 0.95).

ROC analysis revealed that a PFS-HR of 0.60 was a useful cutoff point to distinguish the two types of trial designs, with a sensitivity and specificity of 100% and 100%, respectively, and an area under the ROC curve (AUC) of 1.00 (Table 3, Fig. 4A).

**Table 3:** The accuracy by which molecularly selected patient trials can be distinguished from all-comer trials using two alternative endpoints.

Endpoint with the most accurate cutoff level		Molecularly selected patient trials (*n* = 8)	All-comer trials (*n* = 10)	Sensitivity	Specificity	AUC
PFS-HR				100%	100%	1.00
	< 0.60	8	0			
	≥ 0.60	0	10			
Odds ratio for the overall response				88%	90%	0.95
	≥ 3.40	7	1			
	< 3.40	1	9			
Both the PFS-HR and odds ratio for the overall response						
	< 0.60 and ≥ 3.40	7	0			
	Other	1	10			
Either the PFS-HR or the odds ratio for the overall response						
	< 0.60 or ≥ 3.40	8	1			
	Other	0	9			

AUC, area under the receiver operating characteristic curve.

### Odds ratios of the overall response both in molecularly selected patient and all-comer trials

The odds ratio of the overall response was higher in trials with molecularly selected patients than in all-comer trials (mean: 6.10 vs. 1.64, respectively; *P* < 0.01, Fig. 3C). This was maintained upon multivariate analysis; the former trial type had a 4.46-point greater odds ratio than that of the all-comer trial; *P* < 0.001; Table 2).

The ROC curve indicated that an odds ratio of 3.40 for the overall response was a potentially useful cutoff point to identify trials with molecularly selected patients, affording a sensitivity of 88%, a specificity of 90%, and an AUC of 0.95 (Table 3, Fig. 4B). The odds ratio, even in combination with the PFS-HR, did not increase the probability of detecting trials of molecularly selected patients (Table 3).

## Discussion

We noted robust benefits in terms of both the PFS and overall response in trials evaluating approved EGFR-TKIs or ALK-TKIs in molecularly selected patients (Fig. 3B and 3C, Table 3). In particular, a PFS-HR of approximately 0.6 was a useful cutoff for distinguishing molecularly selected patient trials from all-comer trials, with a sensitivity of 100% and a specificity of 100% (Fig. 4A, Table 3). To date, PFS has not been shown to be a statistically acceptable surrogate for OS because of the lack of a strong association between PFS and OS in advanced NSCLC patients [7,9]. Thus, PFS is not a formal surrogate endpoint but rather a potential future indicator of the clinical benefit of molecular targeted agents in trial designs in which an OS endpoint is of limited utility.

The principal result of our study was that of the 18 phase 3 trials assessing EGFR-TKIs and an ALK-TKI, we found that the PFS-HR yielded by the approved molecular targeted agents in molecularly selected patient trials was sufficiently large to allow distinction between the two trial types, with high accuracy, at a PFS-HR cutoff level of 0.6 (Fig. 3A and Table 3). Assuming that trials with molecularly selected patients have shown and will continue to show only small differences in OS, caused by high levels of crossover treatment, regardless of the effectiveness of the investigational agent [10], the extent of PFS benefit could serve as an important measure of the clinical benefit (Fig. 3A and Table 3). The U.S. Federal Drug Administration (FDA) considers that PFS is a valid clinical endpoint for advanced NSCLC when regulatory decisions on drug approval based on the substantial magnitudes of their effects are to be made [6]. However, “substantial magnitude” remains poorly defined. Here, we describe a cutoff level that will be of potential use in future trials using molecularly selected patients; use of this cutoff will help resolve this long-standing issue.

In contrast, the odds ratio for the overall response seemed less useful for distinguishing trials using molecularly selected patients (Fig. 3C, and Table 3), possibly because the overall response did not accurately reflect dramatic tumor shrinkage; rather, it reflected the proportion of patients in whom the tumor diameter was reduced by ≥ 30%, thus ignoring profound shrinkage [26]. A novel concept is required to establish surrogacy of the overall response; the proportion of patients exhibiting “dramatic responses”, as revealed by a waterfall plot might suffice.

Neither recent randomized trial of LUX-Lung 3 nor-6 revealed any significant OS advantage of afatinib, one of the existing EGFR-TKIs, over the platinum-based chemotherapy, although both combined analysis of these two trials and some subgroup analyses showed an OS benefit of the investigational agent [20]. Our current result would also be applied even in such situation, as long as a trial demonstrates a large PFS benefit but no OS benefit.

A limitation of our study was that all analyses were performed in the absence of any detailed individual patient information, and thus future patient-based data analyses may be necessary to confirm our present findings [27,28]. In addition, we included a limited number of clinical trial analyses that were retrospective in design, and we only analyzed trials evaluating EGFR- or ALK-TKIs. Furthermore, PFS might be a more useful endpoint if it were combined with other endpoints such as quality of life, but no relevant data on this were available to us. Therefore, our work is still at the stage of hypothesis generation; we believe further studies are strongly warranted.

In conclusion, OS is no longer of utility in trials using molecularly selected patients that allow subsequent crossover to active investigational agents. In this situation, these molecularly targeted trials using PFS would be considered positive if their HR is less than or equal to 0.6 for PFS. Although desired threshold might differ in an individual trial, we have contributed critical information to the long-standing debate on potential endpoints alternative to the traditional OS endpoint used in trial design.

## Supporting Information

S1 PRISMA Checklist (DOC)

S1 Table (DOC)
