# Supplementary material for: Magnitude of the Benefit of Progression-Free Survival as a Potential Surrogate Marker in Phase 3 Trials Assessing Targeted Agents in Molecularly Selected Patients with Advanced Non-Small Cell Lung Cancer: Systematic Review
Source: PLoS One. 2015 Mar 16;10(3):e0121211. doi: 10.1371/journal.pone.0121211 (PMC4361736; doi:10.1371/journal.pone.0121211)
Supplement: S1 Table — (DOC) [file pone.0121211.s002.doc]

**Appendix Table A1**

Demographic data from the 18 trials.

| First author | Molecular targeted agents investigated | Year of study initiation | No. of randomized patients | Type of reporting† | Primary endpoint | Line of treatment | Trial design‡ |
| --- | --- | --- | --- | --- | --- | --- | --- |
| Maruyama R | gefitinib | 2003 | 489 | F | OS | 2nd or later | A |
| Kim ES | gefitinib | 2004 | 1466 | F | OS | 2nd or later | A |
| Lee DH | gefitinib | 2005 | 161 | F | PFS | 2nd or later | A |
| Han JY | gefitinib | 2005 | 313 | F | OS | 1st | A |
| Inoue A | gefitinib | 2006 | 230 | F | PFS | 1st | G |
| Mitsudomi T | gefitinib | 2006 | 177 | F | PFS | 1st | G |
| Fukuoka M | gefitinib | 2006 | 1217 | F | PFS | 1st | A |
| Karampeazis A | erlotinib | 2006 | 357 | F | TTP | 2nd or later | A |
| Gridelli C | erlotinib | 2006 | 760 | F | OS | 1st | A |
| Rosell R | erlotinib | 2007 | 174 | F | PFS | 1st | G |
| Garassino MC | erotinib | 2007 | 222 | F | OS | 2nd or later | A |
| Zhou C | erlotinib | 2008 | 165 | F | PFS | 1st | G |
| Yang JC | afatinib | 2009 | 345 | F | PFS | 1st | G |
| Kawaguchi T | erlotinib | 2009 | 301 | F | PFS | 2nd or later | A |
| Yang JC | afatinib | 2010 | 364 | F | PFS | 1st | G |
| Shaw AT | crizotinib | 2010 | 347 | F | PFS | 2nd or later | G |
| Schuler MH | afatinib | 2010 | 202 | A | PFS | 2nd or later | A |
| Mok TS | crizotinib | 2011 | 343 | A | PFS | 1st | G |

†F, full text; A, abstract only; ‡A, all-comer design, G, molecularly selected patient design; OS, overall survival; PFS, progression-free survival; TTP, time to progression.
